# Quantitative clinical assessment of motor function during and following LSVT-BIG® therapy

**DOI:** 10.1186/s12984-020-00729-8

**Published:** 2020-07-13

**Authors:** Matthew W. Flood, Ben P. F. O’Callaghan, Paul Diamond, Jérémy Liegey, Graham Hughes, Madeleine M. Lowery

**Affiliations:** 1 Neuromuscular Systems Lab, School of Electrical & Electronic Engineering, University College Dublin, Belfield, Dublin 4, Ireland; 2 Insight Centre for Data Analytics, O’Brien Centre for Science, University College Dublin, Belfield, Dublin 4, Ireland; 3 Occupational Therapy, Day Hospital, Royal Hospital Donnybrook, Bloomfield Avenue, Dublin 4, Ireland; 4 Department of Geriatric Medicine, St. Vincent’s University Hospital, Elm Park, Dublin 4, Ireland

**Keywords:** Parkinson’s disease, Accelerometry, Exercise therapy, Lee Silverman voice therapy, Gait analysis, Postural control, Wearable sensors, Timed-up-and-go, Sit-to-stand, Finger tapping

## Abstract

**Background:**

LSVT-BIG® is an intensively delivered, amplitude-oriented exercise therapy reported to improve mobility in individuals with Parkinson’s disease (PD). However, questions remain surrounding the efficacy of LSVT-BIG® when compared with similar exercise therapies. Instrumented clinical tests using body-worn sensors can provide a means to objectively monitor patient progression with therapy by quantifying features of motor function, yet research exploring the feasibility of this approach has been limited to date. The aim of this study was to use accelerometer-instrumented clinical tests to quantify features of gait, balance and fine motor control in individuals with PD, in order to examine motor function during and following LSVT-BIG® therapy.

**Methods:**

Twelve individuals with PD undergoing LSVT-BIG® therapy, eight non-exercising PD controls and 14 healthy controls were recruited to participate in the study. Functional mobility was examined using features derived from accelerometry recorded during five instrumented clinical tests: 10 m walk, Timed-Up-and-Go, Sit-to-Stand, quiet stance, and finger tapping. PD subjects undergoing therapy were assessed before, each week during, and up to 13 weeks following LSVT-BIG®.

**Results:**

Accelerometry data captured significant improvements in 10 m walk and Timed-Up-and-Go times with LSVT-BIG® (*p* <  0.001), accompanied by increased stride length. Temporal features of the gait cycle were significantly lower following therapy, though no change was observed with measures of asymmetry or stride variance. The total number of Sit-to-Stand transitions significantly increased with LSVT-BIG® (*p* <  0.001), corresponding to a significant reduction of time spent in each phase of the Sit-to-Stand cycle. No change in measures related to postural or fine motor control was observed with LSVT-BIG®. PD subjects undergoing LSVT-BIG® showed significant improvements in 10 m walk (*p* <  0.001) and Timed-Up-and-Go times (*p* = 0.004) over a four-week period when compared to non-exercising PD controls, who showed no week-to-week improvement in any task examined.

**Conclusions:**

This study demonstrates the potential for wearable sensors to objectively quantify changes in motor function in response to therapeutic exercise interventions in PD. The observed improvements in accelerometer-derived features provide support for instrumenting gait and sit-to-stand tasks, and demonstrate a rescaling of the speed-amplitude relationship during gait in PD following LSVT-BIG®.

## Background

The premise of the speed-amplitude relationship in healthy motor control is that larger amplitude movements are performed at proportionally higher speeds [1, 2]. The speed-amplitude relationship is disproportionately scaled in Parkinson’s disease (PD) [3, 4] and exercise interventions attempting to target this relationship have been shown to encourage natural mobility in individuals with PD [4–6]. One therapy model incorporating this concept is Lee Silverman Voice Therapy – BIG (LSVT-BIG®), developed as a large-amplitude exercise for individuals with PD, incorporating stretching and stepping exercises in combination with functional tasks related to daily living [6–12].

Research evaluating the clinical efficacy of LSVT-BIG® in individuals with PD has thus far been limited [13]. In a study of large amplitude therapy by Farley & Koshland, the first to reference ‘BIG’ exercises, significant increases in gait velocity, stride length, and wrist velocity were observed in 18 PD subjects following therapy [6]. A later study by Ebersbach et al., comparing LSVT-BIG® to a Nordic walking and a home exercise programme, reported significant improvements in MDS-UPDRS III motor scores and Timed-Up-and-Go times, whereas no significant differences were observed in the comparative therapies [9]. In a follow-on study examining cognitive aspects of motor preparation, LSVT-BIG® and Nordic Walking were both shown to improve cued reaction times, but not non-cued reaction times [14]. Despite the various benefits of LSVT-BIG® reported in the literature, issues remain regarding heterogeneity of UPDRS-III scores [13, 15], potentially reflecting the subjectivity or inconsistency of such scores. The disparities in clinical scoring emphasize the need for more objective, quantitative measures of motor function to accurately monitor and assess efficacy of therapeutic interventions in PD.

To obtain a more comprehensive depiction of patient motor function, recent studies have employed body-worn sensors to instrument standard clinical tests, such as the instrumented Timed-Up-and-Go [16–21] and the instrumented Sit-to-Stand [22–24]. These instrumented tests provide an array of quantitative features that can distinguish specific characteristics of motor function in individuals with PD [18, 25–29]. Similarly, studies have used accelerometry (ACC) to analyse postural control in individuals with PD [30–32], and to investigate differences in features of the gait cycle between PD and healthy control subjects, such as step time, swing time and stride time [27, 28, 33, 34]. While these studies highlight the advantages of instrumenting clinical tests to examine patient mobility, there remains a dearth of research incorporating body-worn sensors to monitor and quantitatively assess adaptations in motor function occurring at various stages of physical therapy. Furthermore, few studies have combined a range of instrumented tests to characterise separate aspects of motor function simultaneously.

Against this background, the present work aims to integrate a range of ACC-derived features to extensively examine motor function in individuals with PD, before, each week during, and up to 13 weeks after LSVT-BIG® therapy. Through instrumenting five standard clinical tests – instrumented Sit-to-Stand, instrumented Timed-Up-and-Go, quiet stance, instrumented 10 m walking test and finger tapping – a series of ACC-derived measures are used to examine adaptations in gait, posture, balance and fine motor control, which are then compared with those of healthy age-matched controls and non-exercising PD controls. Overall, this study aims to establish the feasibility of incorporating body-worn accelerometers to objectively monitor and assess adaptions in motor function in individuals with PD following large-amplitude exercise therapy.

## Methods

### Participants

Twelve individuals with idiopathic PD (PD_LSVT_: 9 M, 3F, 74.75 ± 5.91 yrs) were recruited through the local university teaching hospital, where each subject registered to undergo LSVT-BIG®, an amplitude-oriented physical therapy [8]. PD_LSVT_ subjects were bradykinetic-rigid dominant and all remained on their regular levodopa medication schedule throughout therapy. Inclusion criteria for the PD_LSVT_ group included the physical capacity to participate in large-amplitude exercise without frailty, and the cognitive capacity to participate in therapy sessions with functional attention, insight, memory and perceptual ability. Exclusion criteria included any neuromuscular disease other than PD, or impaired cognitive or cardiovascular function preventing them from engaging in large-amplitude exercise. Clinical information regarding PD_LSVT_ subject profiles is provided in Table 1, and a comparison of key clinical measures with previous studies of LSVT-BIG® is provided in the supplementary material, Table S1.

**Table 1:** PD_LSVT_ subject profiles. Levodopa equivalent values (LED) values represent the milligram dosage in each medication consumption

Subject No.	Age	Gender	Height	Weight	BMI	Time Since Diagnosis	Side Most Affected	LED	HY	MDS-UPDRS Total	MDS-UPDRS Motor	PDQ39	ACE III	PAS
*[#]*	*[yrs]*		*[m]*	*[Kg]*	*[Kg/m*^*2*^*]*	*[yrs]*		*[mg]*	*(Baseline)*	*(Baseline)*	*(Baseline)*	*(Baseline)*	*(Baseline)*	*(Baseline)*
1	78	M	1.72	80	26.98	0.5	Right	100^3^	3	58	37	27.56%	79%	8.33%
2	74	M	1.85	123.4	35.89	3.0	Left	100^3^	3	78	42	12.18%	94%	8.33%
3	66	M	1.75	76	24.74	8.0	Right	150^4^	2.5	67	33	26.92%	94%	27.08%
4	76	F	1.65	70.1	25.72	3.2	Right	100^3^	3	40	23	25.00%	96%	16.66%
5	67	M	1.73	72.8	24.25	2.0	Right	100^3^	2	29	9	21.15%	88%	20.83%
6	86	M	1.70	79.1	27.52	0.4	Right	62.5^2^	1.5	16	11	12.82%	88%	6.25%
7	72	F	1.55	67.7	28.20	0.3	Right	12.5^3^	1	9	5	11.54%	88%	18.75%
8	72	M	1.82	80.9	24.36	10.0	Left	100^4^, 200^N^	1.5	41	21	22.44%	83%	4.12%
9	75	F	1.57	66.2	26.69	0.4	Right	62.5^3^	2.5	49	32	12.17%	78%	8.33%
10	84	M	1.80	68.5	21.06	4.0	Left	260^2*^	2.5	38	30	0.00%	87%	0.00%
11	72	M	1.73	74.8	25.07	3.0	Right	0	2.5	65	36	21.13%	87%	41.66%
12	75	M	1.71	109.4	37.49	0.7	Left	150^3^	1.5	35	18	10.26%	90%	25.00%
**Mean**	**74.75**		**1.72**	**80.74**	**27.33**	**2.96**			**2.21**	**43.75**	**24.75**	**16.93%**	**87.67%**	**15.45%**
***(SD)***	***5.91***		***0.09***	***17.61***	***4.77***	***3.14***			***0.69***	***20.72***	***12.11***	***0.08***	***0.06***	***0.12***

2 - BDS, 3 - TDS, 4 - QDS, N - Nocte, * - Controlled Release form

[HY = Hoehn Yahr; MDS-UPDRS = Movement-Disorder-Society Unified-Parkinson’s-Disease-Rating-Scale; PDQ39 = Parkinson’s Disease Questionnaire 39; ACE III = Addenbrooke’s Cognitive Examination; PAS = Parkinson’s Anxiety Scale]

Two control groups consisting of eight individuals with PD not receiving physical therapy (CON_PD_: 5 M, 3F, 78.13 ± 4.85 yrs) and 14 healthy age-matched control subjects (CON_Health_: 9 M, 5F, 70.2 ± 5.85 yrs) were also recruited to perform the same experimental protocol. CON_PD_ subjects were recruited through the same hospital as the PD_LSVT_ group and screened according to the same criteria. CON_Health_ subjects were screened for exclusion criteria which included any major medical conditions, including neurological, neuromuscular or musculoskeletal disorders. Clinical information regarding CON_PD_ subject profiles is provided in the supplementary material, Table S2.

The research protocol was reviewed and approved by the local research ethics committees. All participants provided informed, written consent prior to participation, and all recordings were conducted in accordance with the Helsinki Declaration.

### Intervention

PD_LSVT_ subjects received 16 individual one-hour face-to-face therapy sessions (4 sessions per week for 4 weeks), delivered by an LSVT-BIG® certified occupational therapist. Each hour-long therapy session took place in the morning, Monday to Thursday. The first half of each session involved repeated whole-body drills including sustained large-amplitude stretches and repetitive multidirectional movements. Throughout their programme, subjects were instructed to perform at 80% of their perceived maximum effort, and to provide consistent perceptual feedback as a way of attending to their level of exertion. During the second half of each session subjects performed functional tasks chosen by the subject, such as buttoning a shirt, donning a jacket, or playing a piano, using perceived higher effort levels [6]. Participants were encouraged to adhere to a daily homework schedule, undertaken once on the same day as the treatment session, and twice on days without. The supplementary homework protocol consisted of four repetitions of each core LSVT- BIG® exercise, and practice of functional tasks identified by the therapist.

### Data acquisition

**Fig. 1:**
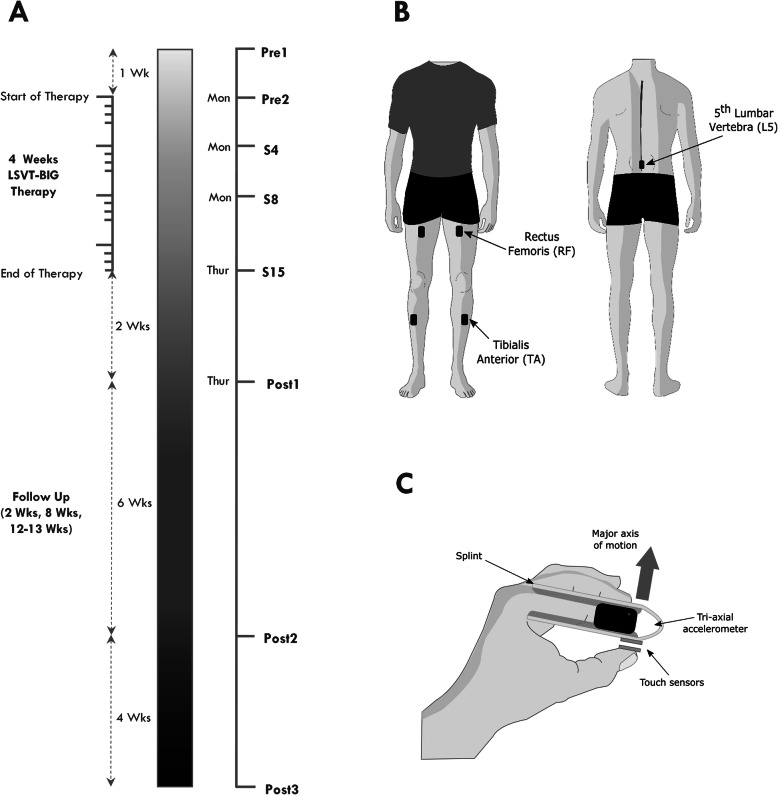
**a** Timeline showing each data recording session before, during and after LSVT-BIG® therapy. Therapy sessions took place each morning Monday to Thursday throughout the 4 weeks of LSVT-BIG®. Data recording sessions Pre2, S4, S8 and S15 took place immediately before LSVT-BIG® therapy sessions 1, 4, 8 and 15 respectively. **b** Position of ACC sensors for the i10MW, iTUG, iSTS and QS tasks. (C) Position of ACC sensor along the medial phalanx of the index finger during the FT task

PD_LSVT_ subjects completed 8 data recording sessions before, each week during and up to 13 weeks after completing therapy (Fig. 1a). Data recording during the course of LSVT- BIG® took place in the morning immediately before the one-to-one therapy session. Follow-up data recording sessions took place on Thursday afternoons at 2 weeks, 8 weeks and 13 weeks post therapy, Fig. 1. The schedule of data recording sessions was consistent across all PD_LSVT_ subjects, outlined in Table S3 in the supplementary material. Each recording session consisted of five instrumented clinical tests of mobility and motor function– instrumented Sit-to-Stand (iSTS), instrumented Timed-Up-and-Go (iTUG), quiet stance (QS), instrumented 10 m walking test (i10MW) and finger tapping (FT). Each test was conducted twice, and subjects were familiarized with the test protocol beforehand. To determine whether weekly repetition of the experimental protocol may influence the performance of PD_LSVT_ subjects, data were recorded from CON_PD_ subjects at four stages (once session per week for 4 weeks) matching the timeline of LSVT- BIG® therapy. CON_Health_ subjects underwent one session. The same setting was used for all sessions of the PD_LSVT_ and CON_PD_ groups.

For the i10MW, iTUG, iSTS and QS tasks, 5 tri-axial accelerometers (Trigno, Delsys Inc., Natick, MA, USA) were positioned on the lower back above the L5 vertebra and on both legs, above the rectus femoris (RF) and tibialis anterior (TA), Fig. 1b. For the FT task, a single accelerometer was placed laterally along the medial phalanx of the index finger where a cushioned, lightweight metal clasp was placed to stabilise it, Fig. 1c. Each sensor weighed 14.7 g, and had dimensions: 37 mm × 27 mm × 15 mm. Accelerometer measurement range and resolution were adjusted for individual tests as outlined in Table 2 and the data were recorded at the default sensor sampling frequency of 148.15 Hz, which could not be adjusted.

**Table 2:** Range and resolution settings for accelerometers in each task

i10MW / iTUG / iSTS	***Locations***	***L5***	***RF/TA***
	Range (g)	±6	±4
	Resolution (g/bit)	0.016	0.0105
**QS**	Range (g)	±1.5	±1.5
	Resolution (g/bit)	0.004	0.004
**FT**	***Locations***	***IF***	
	Range (g)	±4	
	Resolution (g/bit)	0.0105	

### Data analysis

In each test, several features derived from ACC signals were chosen to capture various aspects of motor function. The process of estimating these features is described in the following subsections with a list of all features presented in Table 3.

**Table 3:** List of standard clinical measures and ACC sensor-derived measures with their respective statistical results calculated for each task. Numbers listed in bold font indicate significant *p*-values for one-way RANOVA (α = 0.05) and multiple comparisons with CON_Health_ at stages Pre1, S15 and Post3 (α = 0.017) using student’s two-sample T-test

Task	Feature		Unit	RANOVA	Multiple Comparison w/ CON_**PD**_		
					***Pre1***	***S15***	***Post3***
**i10MW**	Measured Time		s	**< 0.001**	0.545	0.023	0.029
	Gait Times	*Step*	s	**< 0.001**	0.584	0.125	0.061
		*Stride*	s	**< 0.001**	0.525	0.143	0.070
		*Swing*	s	**< 0.001**	0.292	0.395	0.153
		*Stance*	s	**0.037**	0.787	0.091	0.095
	Gait Asymmetry		a.u.	0.932	0.973	0.896	0.728
	Gait Regularity		a.u.	0.317	0.640	0.639	0.179
	Step Time Var		s	0.567	0.730	0.313	0.303
**iTUG**	Measured Time		s	**< 0.001**	0.117	0.355	0.197
	Step Count		#	**0.011**	0.066	0.465	0.163
	Time to First Step		s	0.501	0.582	0.376	0.227
	Gait Times	*Step*	s	**< 0.001**	0.926	0.059	**0.013**
		*Stride*	s	**< 0.001**	0.849	0.066	**0.011**
		*Swing*	s	**< 0.001**	0.689	0.120	0.017
		*Stance*	s	**0.004**	0.515	0.163	0.165
**iSTS**	STS Count		#	**< 0.001**	**0.006**	0.372	0.465
	STS Times	*Total*	s	**0.001**	0.025	0.946	0.193
		*Sit-to-Stand*	s	**< 0.001**	**0.014**	0.823	0.770
		*Stance*	s	**0.011**	0.058	0.814	0.244
		*Stand-to-Sit*	s	**< 0.001**	0.029	0.931	0.051
		*Sit*	s	**0.046**	0.107	0.343	0.617
	Average Jerk		m/s^3^	**0.032**	0.321	0.800	0.226
	STS Variance		a.u	0.092	0.073	0.936	0.253
**QS**	Total Displacement		mm	0.509	0.063	0.081	0.081
	Standard Deviation	*AP*	m/s^2^	0.324	0.129	0.142	0.088
		*ML*	m/s^2^	0.587	0.112	0.204	0.197
	Range	*AP*	m/s^2^	0.315	0.163	0.113	0.108
		*ML*	m/s^2^	0.704	0.451	0.441	0.365
	Normal Path Length	*AP*	m/s	0.117	0.220	0.026	**0.003**
	Jerk		m^2^/s^5^	0.838	0.296	0.245	0.338
**FT**	FT Count		#	0.424	0.819	0.962	0.887
	Intertap Interval		s	0.430	0.761	0.497	0.857
	Kurtosis		a.u.	0.492	0.098	0.226	0.284
	Skewness		a.u.	0.538	0.159	0.881	0.495
	10s/30s Count		a.u.	0.379	0.957	0.445	0.324

### Instrumented 10 m walk (i10MW)

Features of the gait cycle – swing, step, stride and double stance times [35] – were calculated using initial contact and final contact events identified from acceleration signals using the TK_GED_ algorithm [36]. First, the acceleration signal recorded at the shank in the anterio-posterior plane was transformed using the Teager-Kaiser energy operator to capture instances of simultaneous increases in amplitude and frequency. A two-step peak-finding method was subsequently applied to identify initial and final contact events. Measures of gait asymmetry and regularity were estimated using a version of the method proposed by Moe-Nilssen and Helbostad [37] adapted for acceleration recorded from both legs, rather than acceleration recorded at the waist. Acceleration along each axis was used to calculate a resultant magnitude vector, which was then low-pass filtered with a 6th order zero-phase elliptical filter with a 50 Hz cut-off frequency. Unbiased cross-covariance, *Φ*_*LR*_, was calculated between the filtered signals of the left and right legs,

1 $$ {\varPhi}_{LR}(m)=E\left\{{\left({L}_{n+m}-{\mu}_L\right)\left({R}_{n+m}-{\mu}_R\right)}^{\ast}\right\} $$

where *E* is the expected value, *m* is the lag is seconds, *L*_*n*_ and *R*_*n*_ are the filtered left and right leg acceleration vectors respectively, and *μ* is the mean of those vectors. The cross-covariance estimate was normalized by dividing *Φ*_*LR*_ by the square root of the product of the variance of the left, *σ*_*L*_^*2*^, and right, *σ*_*R*_^*2*^, acceleration vectors,

2 $$ {\varPhi_{LR}}^{\ast }=\raisebox{1ex}{${\varPhi}_{LR}$}\!\left/ \!\raisebox{-1ex}{$\sqrt{\sigma_L^2{\sigma}_R^2}$}\right. $$

The value of the first positive-lag local maxima, *M+*, and first negative-lag local maxima, *M-*, about zero were considered to represent the degree of similarity between sequential contralateral steps. Asymmetry was approximated by averaging these values, i.e. $\frac{M_{-}+{M}_{+}}{2.}$

To estimate gait regularity, *Reg*, the unbiased auto-covariance of both legs was calculated using *L*_*n*_ and *R*_*n,*_ and normalized by dividing by the signal variance. The value of the first local maxima, corresponding to the average stride duration, was determined as the measure of inter-stride similarity of each leg – {*M*_*L*_, *M*_*R*_}. A normalised value of gait regularity was estimated from both legs as,

3 $$ Reg=\left|\ln \left(\frac{M_L}{M_R}\right)\right| $$

where values closer to zero indicate higher regularity.

### Instrumented timed-up-and-go (iTUG)

**Fig. 2:**
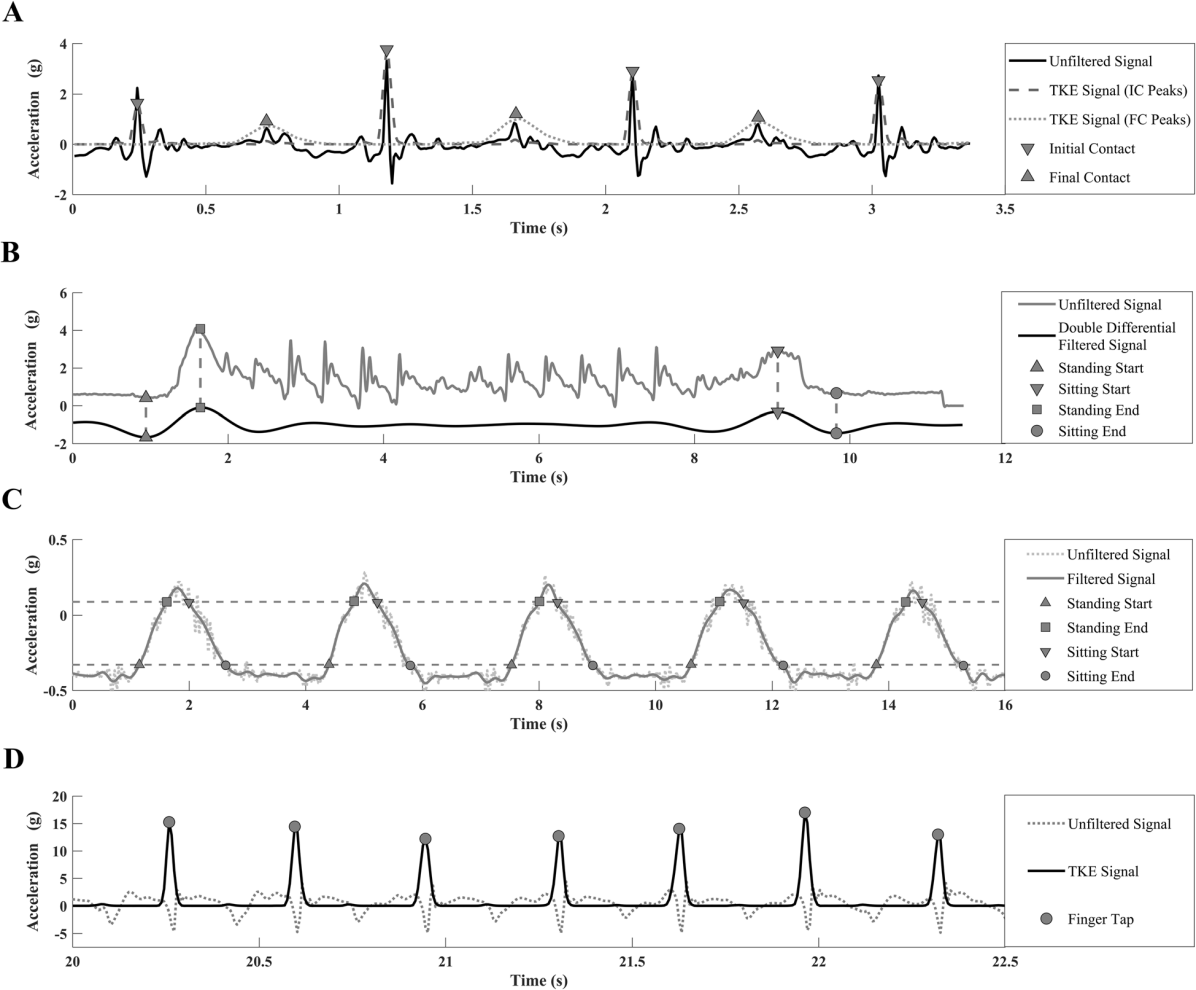
Representative plots of the signal processing steps for deriving features of the iTUG, iSTS and FT tasks. In each subplot, vertical axis units correspond to the raw acceleration signal only. Other signals plotted have been rescaled for visualisation purposes. **a** The acceleration signal along the AP axis as recorded at the shank. Dashed and dotted lines represent the ACC signal processing steps for initial and final contact respectively, using the TK_GED_ algorithm [36]. **b** The acceleration signal recorded from the L5 sensor during the iTUG task. The start and end of the standing and sitting phases were identified as the local minima and maxima respectively of the double-differentiated filtered signal. **c** Events of the Sit-to-Stand cycle used to partition standing, sitting, complete stance and complete sit phases. ACC recorded from the RF was filtered and thresholds for standing and sitting were estimated (black dashed lines). **d** ACC recorded from the medial phalanx of the index finger during the FT task. Finger taps were identified as the local maxima of the ACC signal after filtering using the maximum-overlap discrete wavelet transform followed by the symmetric Teager-Kaiser Energy Operator

Features of the gait cycle during the iTUG were identified using the same method for the i10MW test. In addition to the conventional Timed-up-and-Go measure recorded by the assessor using a stopwatch, the exact time taken to perform the task was estimated from the accelerometer positioned above the L5 vertebra. Acceleration in the AP direction was zero-phase filtered at 0.5 Hz using a 6th order low-pass elliptical filter. The filtered signal was then double differentiated and inverted (Fig. 2b). The start of the standing phase, *T*_*start*_, was approximated as the first local minima, and the end of the standing phase, *T*_*end*_, was the following local maxima. Similarly, the sitting phase was approximated in the same manner. After the standing and gait events were determined, the time to first step was calculated by subtracting *T*_*start*_ from the first heel strike.

### Instrumented sit-to-stand (iSTS)

Acceleration (AP direction at stance) recorded at the RF was low-pass filtered at 8 Hz using a Chebyshev type 2 zero-phase filter. Sit-to-Stand event times, root mean squared jerk, and variance were derived from Sit-to-Stand cycles using a modified method of that reported by Doheny et al. [23], described as follows. The difference, *A*_Δ_, between the median local maxima, *A*_*max*_, and median local minima, *A*_*min*,_ was calculated (Fig. 2c)*.* A Sit-to-Stand transition was defined as beginning when the signal crossed the threshold, T_o_ = *A*_*max*_ – .15*A*_Δ_, and finished when it crossed a second threshold, T_i_ = *A*_*min*_ + .15*A*_Δ_. Similarly, Stand-to-Sit transitions began and finished at T_o_ and T_i_ respectively. The average time spent in standing transition, sitting transition, complete stance, and complete sitting were estimated across all Sit-to-Stand cycles.

To compare iSTS results with those of the five-times Sit-to-Stand test, and for consistent comparison between subjects and stages of therapy, the average time, root mean squared jerk [38], and variance were estimated only from the first 5 cycles. These measures were calculated as the average of the first 5 cycles, with the exception of PD_LSVT_ Subject 1 at Pre1 where the average of all 4 cycles was used, the most complete sit-to-stand cycles Subject 1 could perform at that stage.

### Quiet stance task (QS)

Medio-lateral (ML) and AP acceleration from the L5 sensor were used to evaluate postural control using five measures – standard deviation, root mean squared jerk [38], normalized path length [39], acceleration range, and total displacement [26]. Acceleration offset due to postural misalignment was corrected following the method described by Moe-Nilssen [40]. Artefacts due to noise and tremor were removed by eliminating the first 4 intrinsic mode functions of the empirical mode decomposition [26], equivalent to low-pass filtering at approximately 3.5 Hz. The displacement along each axis was estimated by a further filtering procedure, using a subjective low-pass filter modelled on an inverted pendulum, where the transfer function and corner frequency were estimated for each subject individually as follows [26],

4 $$ H\left( j\omega \right)=-\frac{1}{g+h{\omega}^2},\kern1em {\omega}_n=\sqrt{\frac{g}{h}} $$

where *H(j*ω*)* is the filter transfer function, *g* is the gravitational constant, ω_n_ is the corner frequency, and *h* is the height of the sensor estimated using anthropomorphic scaling of subject height [41, 42]. One second of data following the transition from sitting to stance, estimated using the same method for iTUG, was discarded and all measures were calculated using the subsequent 30s. Due to instrumentation error, QS measures from subjects 1–4 were excluded from the analysis.

### Finger tapping task (FT)

Total finger tapping count and average inter-tap interval values were used to assess fine motor control, while the ratio of taps in the first 10 s to the last 10 s was calculated as an estimate of pacing and rhythm regulation. The skewness and kurtosis of the acceleration signal was also calculated as an indicator of the range of finger motion and tapping acceleration. Instances of each finger tap were obtained using a custom designed algorithm [43]. Acceleration recorded in the vertical axis (perpendicular to the medial phalanx) along the plane of motion of the index finger was decomposed using a maximum overlap discrete wavelet transform with a ‘Haar’ wavelet, and a multiresolution analysis was performed on the wavelet coefficients (equivalent to zero-phase filtering). The output of the multi-resolution analysis was transformed using the Teager-Kaiser energy operator to emphasize the simultaneous changes in amplitude and frequency that occur upon finger-thumb contact [44] (Fig. 2d). A 3-sample moving maximum window, followed by a 5-sample moving mean window was applied to smooth the Teager-Kaiser energy signal. Finally, a peak finding method was used to identify local maxima corresponding to the time instances of each tap (Fig. 2d).

Filtering parameters used in the calculation of each ACC measure were chosen to capture the frequency content of the movement being analysed, while removing noise, and were based on values outlined in the source literature for each ACC feature, where applicable. The processing of all data was performed offline using custom designed algorithms in MatLab 2018 (Mathworks, Natick, MA, USA). A diagrammatic outline of the algorithms used in the derivation of all ACC features is provided in the supplementary material, Figs. S1, S2, S3, S4 and S5.

### Statistical analysis

To assess the effect of LSVT-BIG® on each outcome measure, one-way repeated measures analysis of variance (RANOVA) was performed across all 8 stages. Data sphericity was checked using Mauchly’s test, and the Greenhouse-Geisser correction was applied if sphericity was violated. To determine if the mean of each measure differed significantly throughout therapy, post-hoc analysis with multiple comparisons was performed with Tukey-Kramer correction. Outcome measures of the PD group before therapy (Pre1), at the end of therapy (S15), and at 13 weeks follow-up (Post3) were compared with CON subjects using student’s t-test with an adjusted Bonferroni correction for multiple comparisons (α = 0.017) [45]. To determine whether significant changes in outcome measures of the PD_LSVT_ group would otherwise occur in a non-exercising PD cohort performing the same weekly experiment, a mixed ANOVA was performed on the main clinical score of the i10MW, iTUG and iSTS tasks. For each dependent variable (i10MW time, iTUG time, iSTS count) the mixed ANOVA was modelled with subject group (PD_LSVT_; CON_PD_) as the between-subjects factor and stage of therapy as the within-subjects factor (PD_LSVT_: Pre2 – S15; CON_PD_: Wk1 – Wk4). The interaction of between- and within-subjects factors (group*stage) was evaluated following Greenhouse-Geisser correction wherever sphericity was violated. Dependent variables were normalised with respect to the mean of Pre2 and Wk1 in PD_LSVT_ and CON_PD_ groups, respectively, to compare changes relative to baseline. Statistical analysis was implemented using MatLab 2018a (MathWorks, USA) and SPSS 24 (IBM, USA).

## Results

**Fig. 3:**
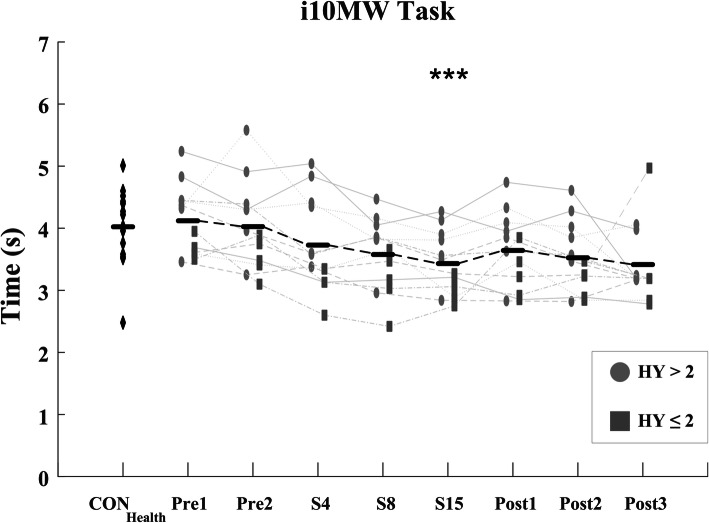
i10MW times for CON_Health_ and PD_LSVT_ subjects at each stage of therapy. i10MW time is the time taken to walk between the 2 m and 8 m mark. Significant improvements in i10MW time occurred in PD_LSVT_ subjects with therapy (*p* < 0.001). Black horizontal lines represent the mean of all PD_LSVT_ subjects at each stage of therapy. (*** *p* < 0.005 compared to baseline - Pre1)

**Fig. 4:**
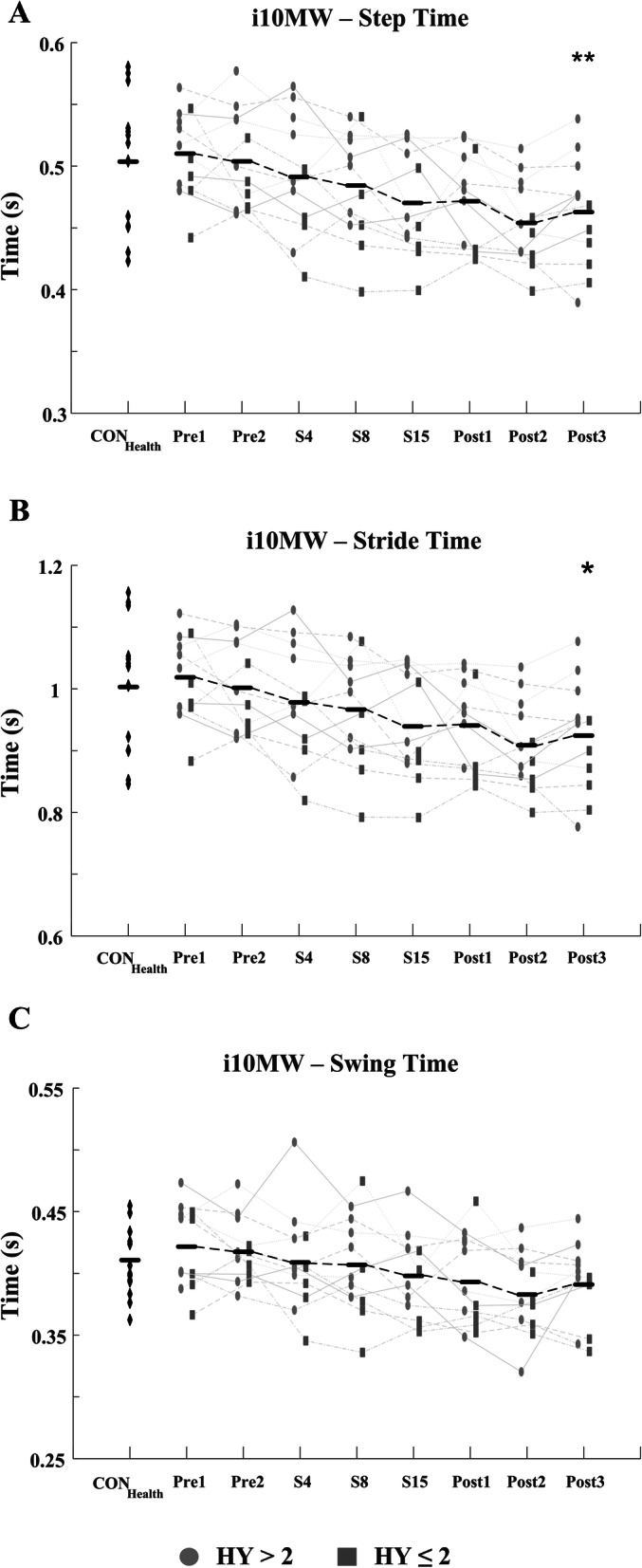
Average i10MW (**a**) step, (**b**) stride and (**c**) swing times for CON_Health_ and PD_LSVT_ subjects at each stage of therapy. PD_LSVT_ subjects developed significantly faster step (*p* < 0.001), stride (*p* < 0.001), swing (p < 0.001), and double stance times (*p* < 0.037) following therapy. Black horizontal lines represent the mean of all PD_LSVT_ subjects at each stage of therapy. (** *p* < 0.01, * *p* < 0.05 compared to baseline - Pre1)

**Fig. 5:**
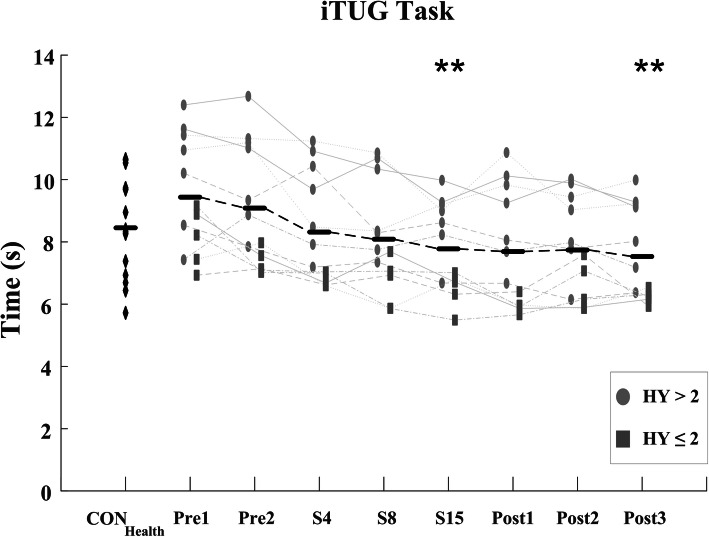
Overall iTUG time for CON_Health_ and PD_LSVT_ subjects at each stage of therapy. iTUG time is the time taken to stand up, walk 3 m around a cone and return to the seated position. Significant improvements in iTUG time occurred in PD_LSVT_ subjects with therapy (*p* < 0.001). Black horizontal lines represent the mean of all PD_LSVT_ subjects at each stage of therapy. (** *p* < 0.01 compared to baseline - Pre1)

**Fig. 6:**
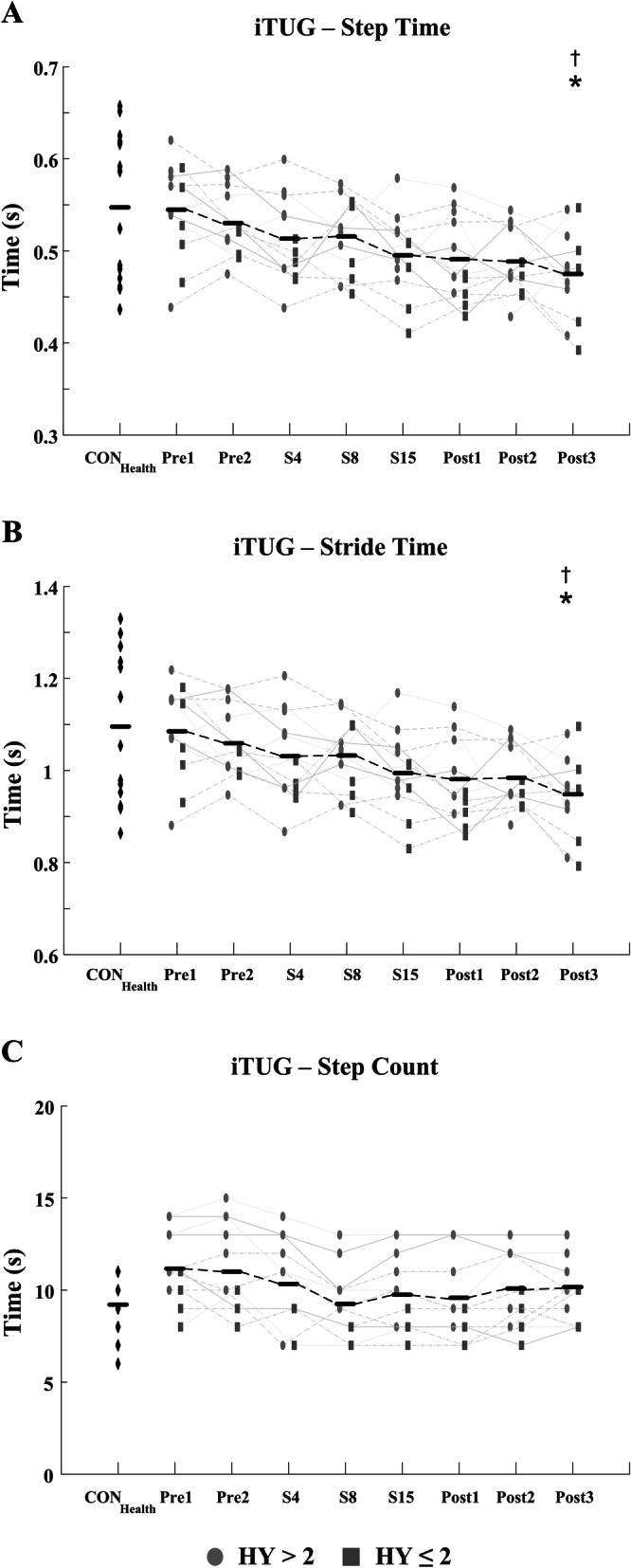
Estimated (**a**) step time, (**b**) stride time and (**c**) step count of iTUG task for CON_Health_ and PD_LSVT_ subjects at each stage of therapy. PD_LSVT_ subjects developed significantly faster step (*p* < 0.001), stride (*p* < 0.001), swing (*p* < 0.001), and double stance times (*p* < 0.004) following therapy. In addition to temporal gait parameters, significant reductions were observed in step count following therapy (*p* < 0.011) indicative of longer step lengths. Black horizontal lines represent the mean of all PD_LSVT_ subjects at each stage of therapy. (* *p* < 0.05 compared to baseline - Pre1; † *p* < 0.05 compared to CON_Health_)

Significant improvements in the i10MW walk time (F = 5.88; *p* <  0.001) and iTUG time (F = 13.94; *p* <  0.001) were observed in the PD_LSVT_ cohort over the course of LSVT-BIG® therapy, Figs. 3 & 5 and Table 3. Temporal parameters of the gait cycle steadily decreased throughout therapy in both the i10MW and iTUG tasks, with significant reductions observed in step time (i10MW: F = 9.12, *p* <  0.001; iTUG: F = 7.54, *p* <  0.001), stride time (i10MW: F = 8.63, *p* <  0.001; iTUG: F = 7.03, *p* <  0.001), swing time (i10MW: F = 5.34, *p* <  0.001; iTUG: F = 4.31, *p* <  0.001) and stance time (i10MW: F = 3.22, *p* = 0.037; iTUG: F = 3.23, *p* = 0.004), Fig. 4. Post-hoc analysis showed that significantly lower gait times predominantly occurred at follow-up stages (Post1 – Post3) when compared to pre-therapy (Pre1, Pre2), Figs. 4 & 6. No effect was observed for the stage of therapy on the gait asymmetry (*p* = 0.93), gait regularity (*p* = 0.32), or step time variance (*p* = 0.56) measures. While the time to first step did not change in the iTUG task (*p* = 0.501), the total step count significantly decreased with therapy (F = 2.86, *p* <  0.011), Fig. 6.

**Fig. 7:**
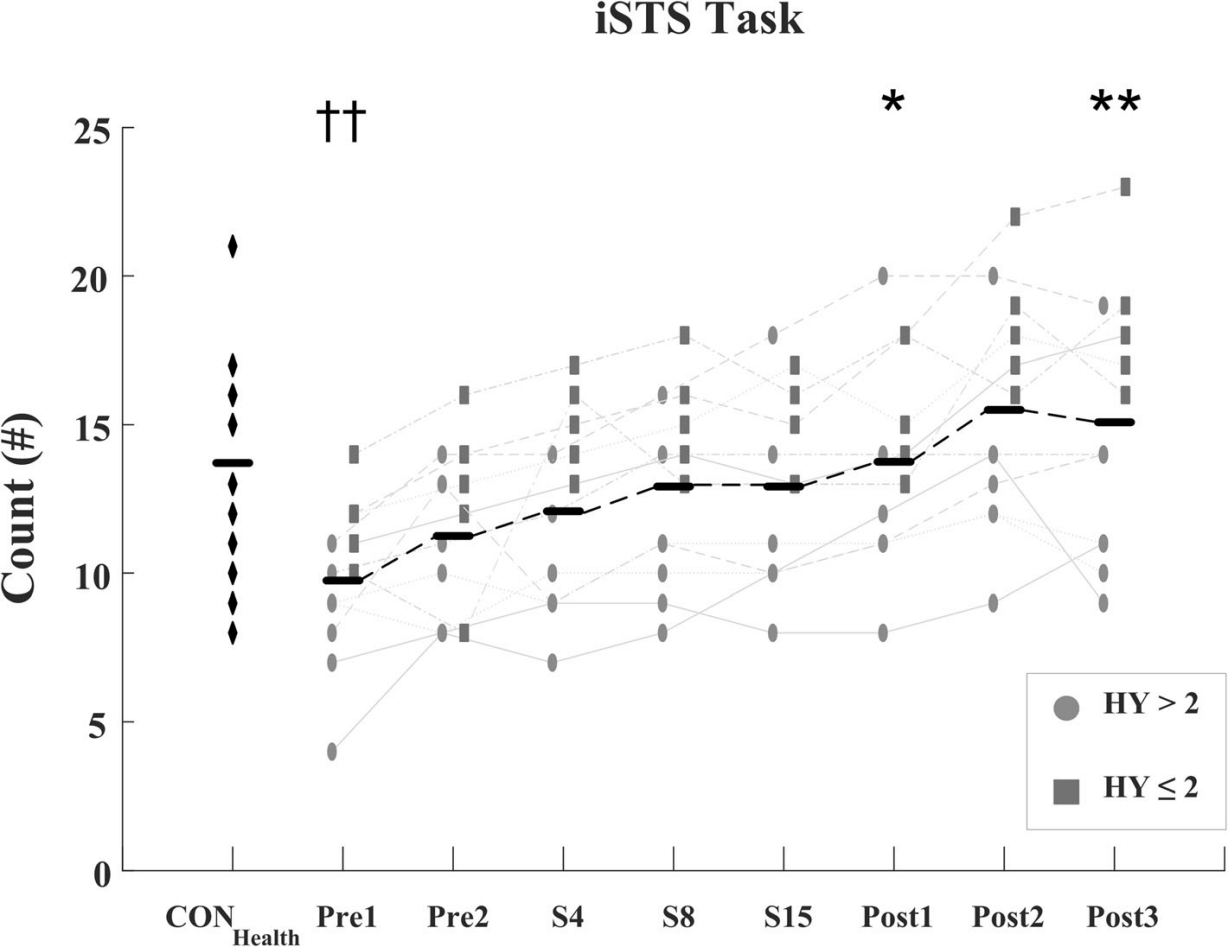
iSTS count for CON_Health_ and PD_LSVT_ subjects at each stage of therapy. iSTS count is the number of times a subject could stand up and sit down in 30s. Significantly higher counts occurred in PD_LSVT_ subjects with therapy (p < 0.001). Black horizontal lines represent the mean of all PD_LSVT_ subjects at each stage of therapy. (* *p* < 0.05, * *p* < 0.01 compared to baseline - Pre1; †† p < 0.01 compared to CON_Health_)

**Fig. 8:**
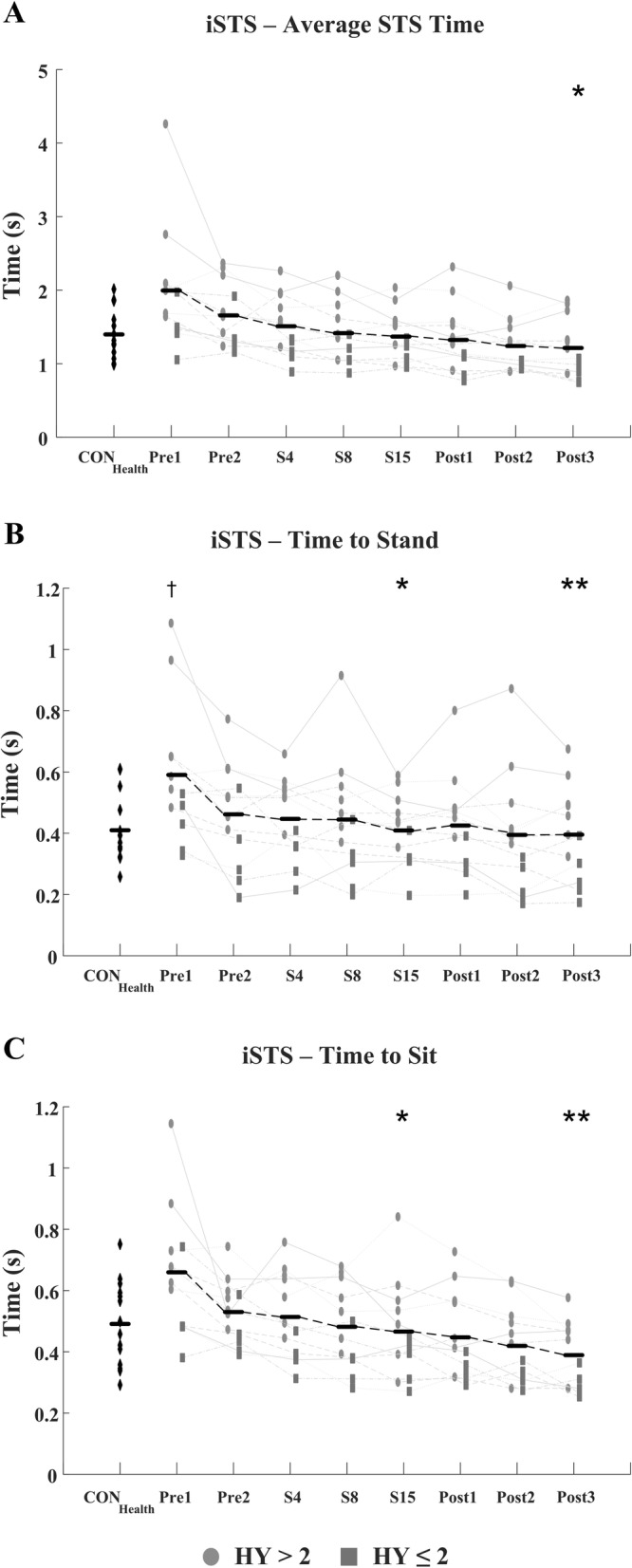
Estimated (**a**) average total STS time, (**b**) standing time and (**c**) sitting time of the iSTS task for CON_Health_ and PD_LSVT_ subjects at each stage of therapy. PD_LSVT_ subjects developed significantly faster times with therapy for each temporal feature examined (*p* < 0.001). Black horizontal lines represent the mean of all PD_LSVT_ subjects at each stage of therapy. (* *p* < 0.05, * *p* < 0.01 compared to baseline - Pre1; † *p* < 0.05 compared to CON_Health_)

The iSTS count also significantly improved with therapy (F = 14.39; *p* <  0.001) matched by a significant reduction in the total average Sit-to-Stand time (F = 10.12, *p* <  0.001), Standing time (F = 8.36, *p* <  0.001), Sitting time (F = 10.06, *p* <  0.001), complete stance time (F = 5.63, *p* = 0.011), and complete sit time (F = 3.73, *p* = 0.046), Figs. 7 & 8 and Table 3. As observed in the i10MW and iTUG tasks, post-hoc comparisons showed significant differences in iSTS measures predominantly occurring between pre-therapy (Pre1, Pre2) and post-therapy (Post2, Post3) stages. The root-mean-square jerk of the first 5 Sit-to-Stand cycles significantly increased with therapy (F = 3.37, *p* = 0.032), whereas no difference was observed in the variance of the first 5 Sit-to-Stand cycles (*p* = 0.092). Despite the improved scores of the iTUG, i10MW, and iSTS tasks, LSVT-BIG® had no effect on any measure in the QS tasks (RMS Jerk: *p* = 0.84; Normalized Path Length: *p* = 0.18; Displacement: *p* = 0.51) or FT tasks (Tap Count: *p* = 0.42; Intertap Interval: *p* = 0.43; 10s/30s Count: *p* = 0.38), Table 3. The daily levodopa equivalent dosage (LED) did not change for any PD_LSVT_ subject during therapy, Table 1.

Total iSTS count was significantly lower (*p* = 0.006), and Sit-to-Stand time significantly higher (*p* = 0.014), in the PD_LSVT_ group at baseline than in the CON_Health_ group, Table 3. Following therapy, both measures converged towards values similar to those of CON_Health_, Figs. 7 & 8. The temporal parameters of the gait cycle in the PD_LSVT_ group were comparable with those of CON_Health_ at baseline, with step and stride time significantly lower after therapy (Post3 - step time: *p* = 0.013, stride time: *p* = 0.011), Figs. 4 & 6 and Table 3.

**Fig. 9:**
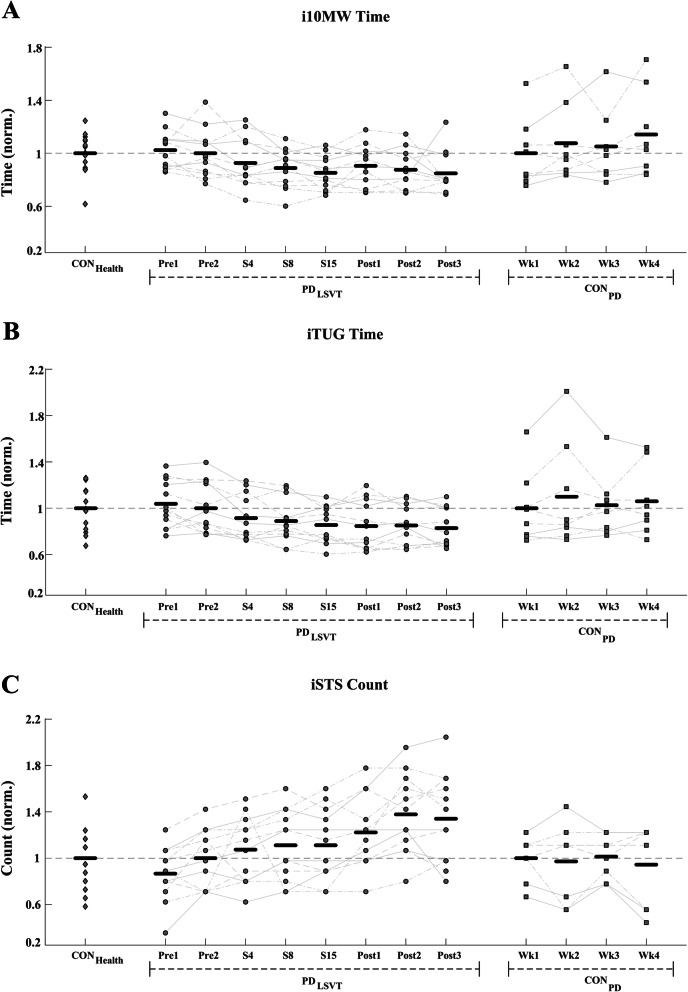
(**a**) i10MW time, (**b**) iTUG time (**c**) iSTS counts for CON_Health,_ PD_LSVT_ and CON_PD_ subjects. To control for inter-group variation at baseline, values displayed are normalised with respect to the mean CON_Health_ value, mean PD_LSVT_ value at Pre2, and mean CON_PD_ value at Wk1, for each group respectively. In contrast to PD_LSVT_ subjects undergoing therapy, CON_PD_ subjects performing the same experimental protocol over a four-week period did not show a consistent improvement in the (**a**) i10MW, (**b**) iTUG, or (**c**) iSTS tasks. Results of the mixed-model ANOVA indicate that subjects participating in LSVT- BIG® achieve significantly better scores than a non-exercising control group in the i10MW (*p* < 0.001) and iTUG (*p* = 0.004) tasks over a four-week period

When compared over a four-week period from the beginning of therapy (Pre2), PD_LSVT_ subjects showed a consistent weekly improvement in i10MW, iTUG and iSTS clinical scores, in contrast to the non-exercising CON_PD_ group over the same period, Fig. 9. Results of the mixed ANOVA showed a significant interaction effect between subject group and stage of therapy in the i10MW (F = 9.06, *p* <  0.001) and iTUG (F = 5.10, *p* = 0.004) tasks, indicating that weekly delivery of LSVT-BIG® therapy significantly improves functional mobility when compared to a non-exercising PD cohort examined over the same period. Although no interaction effect was observed for the iSTS count over the period examined, PD_LSVT_ subjects had significantly higher iSTS counts at S15 when compared to CON_PD_ subjects at Wk4 (two-sample T-test: *p* = 0.018). CON_PD_ subjects performing the same experimental protocol showed no within-group change in i10MW time (F = 2.44, *p* = 0.092), iTUG time (F = 1.16, *p* = 0.347) or iSTS count (F = 0.48, *p* = 0.694) over a four-week period, Fig. 9.

## Discussion

The stretching and stepping exercises that LSVT-BIG® incorporates are designed to enhance the speed-amplitude relationship that is disproportionately scaled by PD [4, 5]. By integrating these exercises with functional aspects of daily living, LSVT-BIG® also aims to improve the internal cueing mechanisms that regulate motor control in various environments. While several studies have previously investigated the effect of LSVT-BIG® on motor and non-motor symptoms in PD [6, 9–11, 13, 14, 46, 47], no study has yet quantitatively evaluated motor function using wearable sensors at multiple stages before, throughout, and up to 13 weeks post LSVT-BIG® therapy. Additionally, this study provided an examination of balance, postural control and fine motor function through iSTS, QS and FT tasks respectively, and compared outcome measures from all tasks with data from healthy age-matched controls.

The present study showed that gait speed increased in subjects undergoing LSVT-BIG®, demonstrated by significantly lower i10MW and iTUG times at follow-up compared to baseline (*p* <  0.001; Figs. 3, 4, 5 and 6 and Table 3) and to a non-exercising PD cohort (i10MW: *p* <  0.001; iTUG: *p* = 0.004; Fig. 9). These results are consistent with those reported in previous studies where i10MW and Timed-Up-and-Go times decreased with LSVT-BIG® [6, 9, 13, 14, 47, 48]. By employing wearable sensors to detect gait events, the gait cycle was deconstructed into its constituent time phases, collectively showing significant reductions in step, stride, swing and stance times with therapy (Figs. 4 & 6, Table 3). As these times were also accompanied by significantly lower step counts (*p* = 0.010), these results provide further evidence that PD subjects undergoing LSVT-BIG® develop longer step lengths with faster step time [6, 10], suggesting a rescaling of the speed-amplitude relationship. Despite these outcomes, no change was observed in step time variance, gait asymmetry or gait regularity, measures. These features have been shown to be independent of gait speed [49] and related to attentional demand in PD [50, 51]. While LSVT-BIG® is not specifically designed to address gait variability, these measures are important due to their association with increased risk of falls [27, 50, 51]. The lack of any observable improvement in these measures with therapy may also be confounded by effects due to levodopa, shown to reduce gait variability [52].

Improvements in functional mobility with LSVT-BIG® were observed in the iSTS task, where the number of Sit-to-Stand cycles performed significantly increased with therapy (*p* <  0.001), Fig. 7 and Table 3. Similar to the gait cycle, the duration of each phase of the Sit-to-Stand cycle significantly decreased with therapy (Fig. 8), particularly in the standing and sitting phases where the average time pre-therapy reduced by approximately a third at post-therapy, potentially indicating enhanced muscular strength, greater effort, and improved balance. To the best of the authors’ knowledge, this is the first study to use the iSTS task to assess subject mobility in response to LSVT-BIG®, and as evidenced by the faster Sit-to-Stand times, demonstrates that the benefits of LSVT also translate to the iSTS task.

Although the mean value of i10MW, iTUG and iSTS outcome measures indicated an overall improved performance between Pre1 and Pre2 (Fig. 9), on an individual level this was not necessarily the case. For example, five of the twelve PD_LSVT_ subjects took longer to perform the iTUG task at Pre2 than at Pre1, while a third of subjects had slower step and swing times at Pre2 in the i10MW task. Data collection at Pre2 occurred on the first day of each subject’s LSVT-BIG® therapy. Therefore, it is possible that participants, eager to start therapy, may have been more motivated than usual at Pre2 compared to Pre1. Alternatively, the improvements observed at Pre2 may be associated with familiarisation of the experimental protocol at Pre1. However, it is evident from Fig. 9 that the non-exercising CON_PD_ subjects, performing the same weekly experimental protocol as those in the PD_LSVT_ group, did not show any consistent improvement in their i10MW, iTUG or iSTS clinical scores. This observation was supported by a RANOVA analysis of each clinical score, where no significant weekly change was observed in i10MW time, iTUG time or iSTS count, indicating that practice or familiarisation with the experimental protocol does not by itself influence subject performance on a week-to-week basis. Thus, the changes observed in PD_LSVT_ subject performance from Pre1 to Pre2 were likely due to a separate factor. The average values of the clinical scores for the CON_PD_ subjects were slightly lower than for PD_LSVT_ subjects, HY (1.25 ± 0.46) and MDS-UPDRS (22.75 ± 11.56). To check whether the difference in clinical scores could account for the different progression observed in the two groups, the mixed-ANOVA was reevaluated comparing the CON_PD_ group against the eight PD_LSVT_ subjects with the lowest MDS-UPDRS Total scores. The analysis confirmed a significant interaction effect between subject group and stage of therapy in the i10MW (*p* = 0.001) and iTUG (*p* = 0.011), with no significant difference in clinical scores between groups (MDS-UPDRS Total: *p* = 0.16; MDS-UPDRS III: *p* = 0.19). These results demonstrate that the disparity in initial clinical scores between the PD subject groups does not influence the overall findings of the study.

While the average iSTS count and stand-to-sit transition time were significantly different between CON_Health_ and PD_LSVT_ groups at baseline, the i10MW time, iTUG time, and the respective gait cycle features were initially comparable with CON_Health_ values, a potential consequence of several factors. Firstly, most PD_LSVT_ subjects reported being moderately active, with several walking regularly. Secondly, it has been demonstrated that PD subjects walk faster and with larger steps in the clinical environment due to a ‘performance effect’, where concentration is elevated and peripheral distraction is attenuated [10, 28]. Comparing gait in PD and healthy control subjects, Del Din et al., showed that measures of gait asymmetry and gait rhythm increased in PD subjects under free-living conditions, when no difference between groups was identified under laboratory conditions [28]. Hence, the relatively active nature of the PD_LSVT_ subjects, combined with a potential performance effect in the clinical environment may explain the similar i10MW and iTUG times between the CON_Health_ and PD_LSVT_ groups pre-therapy. An important element of LSVT-BIG® is adherence to a home exercise regimen in addition to one-to-one therapy received within the clinic. The homework protocol consists of four repetitions of each core LSVT-BIG® exercise, as well as large-amplitude walking and practice of functional tasks identified by the therapist. Although beyond the scope of the present study, future studies could extend the data analysis employed here to examine functional mobility from the same tasks performed at home, where the performance effect observed in the clinic may be absent.

In contrast to the large amplitude tests (i10MW, iTUG, iSTS), LSVT-BIG® showed no effect on any measure of the QS or FT tasks. Two meta-analyses of postural stability in PD have suggested that without specific balance-oriented training, only minor improvements in postural stability can be achieved [53, 54]. Furthermore, it has been recommended that a minimum of 8 weeks training is necessary to achieve substantial improvement in balance in PD [55], twice the duration of LSVT-BIG®. Thus, measures of balance during the QS task are unlikely to improve with LSVT-BIG®, as the focus of the therapy is on amplitude and not postural stability. Alternatively, the absence of any significant improvement in QS with LSVT-BIG® may relate to the type of measure, and how it is derived using ACC. Using the Berg Balance Test, Millage et al. have been the only researchers to specifically evaluate postural balance in individuals undergoing LSVT-BIG® therapy, but their findings were limited as four out of nine subjects achieved maximum scores prior to therapy, leaving a sample size of just 5 subjects [47].

Janssens et al., hypothesized that the finger-spreading exercises of LSVT-BIG® may help to improve manual dexterity, evaluated using the nine peg hole test [46]. Although a slight improvement was observed in the non-dominant hand, dexterity in the dominant hand did not benefit from LSVT-BIG®. In contrast, Ebersbach et al. have reported significantly improved manual dexterity with LSVT-BIG®, assessed using the box-block test [10, 56]. Given the lack of any observable improvement in FT measures, it remains unclear whether or not LSVT-BIG® benefits upper limb motor function in PD. However, the disparate type of motion assessed in the nine-peg-hole test, box-block test, and FT tasks may account for the inconsistency among studies. As the goal of LSVT-BIG® is to reinforce large amplitude movements, it is expected that such a therapy will have minimal impact on FT, which involves small amplitude movements, or QS, where the aim of the task is to minimize sway.

Many of the studies reporting on the efficacy of LSVT-BIG® that have been performed to date, although insightful as to the benefits of therapy, are limited by low subject numbers [11, 13, 46, 47], the absence of a PD or healthy control group [6, 9–11, 13, 14, 46, 47], or a lack of detail on specific aspects of kinematic improvements [9–11, 13, 14, 46, 47]. These shortcomings have been commonly observed across a spectrum of physical therapy studies in PD [15, 57], where it is unclear if improved mobility is a result of enhanced muscle mass or whether positive effects wear off post-therapy [13, 58]. The present work has addressed some of these issues by continually recording patients throughout therapy, by quantifying functional mobility at each stage and by comparing results with controls, yet there are several limitations which remain. Firstly, subjects remained on their prescribed levodopa medication which did not change throughout the course of therapy. Levodopa has been shown to affect various aspects of balance [59] and gait [52] in individuals with PD, which may mask the true effect of LSVT-BIG® on parkinsonian symptoms, or introduce behaviour in the ACC signal that obfuscates the measures derived. Secondly, no alternative therapy was examined against which to compare the effect of LSVT-BIG®. Thus, it cannot be stated whether LSVT-BIG® is more beneficial than other exercise-based therapies. However, consistent with the present findings, results from the Berlin BIG [9, 14] and JFK BIG [48] studies have shown LSVT-BIG® to benefit individuals with PD, helping to reduce bradykinesia and ameliorate the speed-amplitude relationship of large-amplitude movement.

## Conclusions

Features derived from body-worn accelerometers showed that subjects undergoing LSVT-BIG® develop enhanced functional mobility which remains sustained up to 13 weeks post therapy. Although no significant effect was observed in measures of postural or fine motor control, temporal parameters of the gait and Sit-to-Stand cycles significantly reduced with therapy. The results indicate that LSVT-BIG® helps to partially restore the speed-amplitude relationship for large amplitude movement, matching that of age-matched controls. These findings are consistent with those of previous studies and extend previous findings by employing wearable ACC sensors to provide insight into specific features of movements performed during clinical tests.

The insights gained from the objective, quantifiable measures can provide clinicians with information about the efficacy of high-amplitude movement therapies, such as LSVT-BIG®, and can be used to target patients with specific subtypes of Parkinson’s Disease that are more likely to respond to a high-amplitude therapy programme.

## Supplementary information

**Additional file 1: Table S1**. Mean values for PD subject characteristics in the present and previous studies on LSVT-BIG®.

**Additional file 2 Table S2**: CON_PD_ subject profiles.

**Additional file 3 Table S3.** Stages of assessment and the corresponding time points at which they occurred.

**Additional file 4 Fig. S1.** Processing steps for ACC-measures calculated in i10MW task.

**Additional file 5 Fig. S2.** Processing steps for ACC-measures calculated in iTUG task.

**Additional file 6 Fig. S3.** Processing steps for ACC-measures calculated in iSTS task.

**Additional file 7 Fig. S4.** Processing steps for ACC-measures calculated in QS task.

**Additional file 8 Fig. S5.** Processing steps for ACC-measures calculated in FT task.

## Data Availability

The datasets used and analysed during the current study are available from the corresponding author on reasonable request.

## Abbreviations

ACC: Accelerometry
ACE III: Addenbrooke’s Cognitive Examination
AP: Anterio-posterior axis
CON_Health_: Healthy Control group
CON_PD_: Parkinson’s Disease Control Group
FT: Finger Tapping test
HY: Hoehn-Yahr Score
i10MW: Instrumented 10 m Walk Test
iSTS: Instrumented Sit-to-Stand Test
iTUG: Instrumented Timed-Up-and-Go Test
L5: Fifth Lumbar Vertebra
LED: Levodopa Equivalent Dosage
LSVT-BIG®: Lee Silverman Voice Therapy – BIG
MDS-UPDRS: Movement Disorders Society Unified-Parkinson’s-Disease-Rating Scale
ML: Medio-lateral axis
PAS: Parkinson’s Anxiety Scale
PD: Parkinson’s Disease
PD_LSVT_: LSVT-BIG Parkinson’s Disease Group
PDQ39: Parkinson’s Disease Questionnaire 39
QS: Quiet Stance Test
RANOVA: Repeated Measures Analysis-of-Variance
RF: Rectus Femoris Muscle
TA: Tibialis Anterior Muscle
