# Supplementary material for: Quantitative clinical assessment of motor function during and following LSVT-BIG® therapy
Source: J Neuroeng Rehabil. 2020 Jul 13;17:92. doi: 10.1186/s12984-020-00729-8 (PMC7359464; doi:10.1186/s12984-020-00729-8)
Supplement: Supplementary file 1 — Additional file 1: Table S1. Mean values for PD subject characteristics in the present and previous studies on LSVT-BIG®. [file 12984_2020_729_MOESM1_ESM.docx]

Table S1: Mean values for PD subject characteristics in the present and previous studies on LSVT-BIG^®^. In contrast to previous reports, symptom severity in the present study was assessed using the MDS-UPDRS*, and Time Since Diagnosis** is reported rather than Disease Duration*

| **Reference Study** | **Subjects**  **#** | **HY** | **MDS-UPDRS* /  UPDRS III** | **LED (mg/day)** | **Time Since Diagnosis** / Disease Duration (yrs)** |
| --- | --- | --- | --- | --- | --- |
| Present Study | 12 | 2.21 | 24.75* | 310 | 2.96** |
| [1] | 6 | 1.3 | 17.2 | N/A | 2.9 |
| [2] | 3 | 1 - 3 | 24.33 | 966 | 5.66 |
| [3] | 20 | 2.8 | 21.1 | 486 | 6.1 |
| [4] | 17 | 1 - 3 | 18.2 | 557 | 4 |
| [5] | 18 | 1.94 | N/A | N/A | N/A |
| [1] Dashtipour *et al.*, *Parkinsons. Dis.*, 2015; 2015:1–5.  [2] Janssens *et al.,* *Phys. Ther.*, 2014; 94(7):1014–1023.  [3] Ebersbach *et al.*, *Mov. Disord.*, 2010; 25(12):1902–1908.  [4] Ebersbach, *et al.*, *J. Neural Transm.*, 2014; 122(2):253–256.  [5] Farley and Koshland, *Exp. Brain Res.*, 2005; 167(3): 462–467. | | | | | |
