# Supplementary material for: Quantitative clinical assessment of motor function during and following LSVT-BIG® therapy
Source: J Neuroeng Rehabil. 2020 Jul 13;17:92. doi: 10.1186/s12984-020-00729-8 (PMC7359464; doi:10.1186/s12984-020-00729-8)
Supplement: Supplementary file 2 — Additional file 2 Table S2: CONPD subject profiles. [file 12984_2020_729_MOESM2_ESM.docx]

Table S2: CON_PD_ subject profiles. Levodopa equivalent values (LED) values represent the milligram dosage in each medication consumption.

[HY = Hoehn Yahr; MDS-UPDRS = Movement-Disorder-Society Unified-Parkinson's-Disease-Rating-Scale; PDQ39 = Parkinson’s Disease Questionnaire 39; ACE III = Addenbrooke’s Cognitive Examination; PAS = Parkinson’s Anxiety Scale]

|  | **Subject  No.** | **Age** | **Gender** | **Height** | **Weight** | **BMI** | **Time  Since  Diagnosis** | **Side  Most Affected** | **LED** | **HY** | **MDS-UPDRS Total** | **MDS-UPDRS Motor** | **PDQ39** | **ACE III** | **PAS** |
| --- | --- | --- | --- | --- | --- | --- | --- | --- | --- | --- | --- | --- | --- | --- | --- |
|  | *[#]* | *[yrs]* |  | *[m]* | *[Kg]* | *[Kg/m^2^]* | *[yrs]* |  | *[mg]* | *(Baseline)* | *(Baseline)* | *(Baseline)* | *(Baseline)* | *(Baseline)* | *(Baseline)* |
| **CON_PD_ Group** | 1 | 77 | M | 1.55 | 70.4 | 45.42 | 2.0 | Left | 50^3^, 100^M^, 20^T^ | 1 | 27 | 8 | 21.15% | 92% | 14.58% |
|  | 2 | 86 | M | 1.64 | 64.1 | 39.09 | 0.6 | Right | 100^3^ | 1 | 16 | 12 | 23.08% | 80% | 16.66% |
|  | 3 | 76 | F | 1.67 | 64.9 | 38.86 | 2.0 | Right | 100^2^, 37.5^*N^ | 1 | 10 | 6 | 11.54% | 94% | 10.42% |
|  | 4 | 75 | M | 1.79 | 86.7 | 48.44 | 2.0 | Left | 0 | 1 | 19 | 13 | 12.82% | 76% | 6.25% |
|  | 5 | 81 | M | 1.75 | 66.4 | 37.94 | 2.0 | Left | 100^3^ | 2 | 17 | 12 | 18.59% | 80% | 10.41% |
|  | 6 | 71 | F | 1.57 | 74.1 | 47.20 | 3.0 | Right | 100^4^, 75^*N^ | 2 | 48 | 27 | 32.05% | 77% | 45.83% |
|  | 7 | 76 | M | 1.77 | 66.5 | 37.57 | 0.6 | Right | 0 | 1 | 26 | 12 | 15.60% | 89% | 8.33% |
|  | 8 | 83 | F | 1.75 | 77.1 | 44.06 | 0.9 | Left | 125^M^, 125^4^ | 1 | 19 | 10 | 19.23% | 88% | 25.00% |
|  | **Mean** | **78.13** |  | ***1.69*** | ***71.28*** | ***42.32*** | ***1.64*** |  |  | ***1.25*** | ***22.75*** | ***12.50*** | **19.26%** | **84.50%** | **17.19%** |
|  | ***(SD)*** | **4.85** |  | ***0.09*** | ***7.74*** | ***4.44*** | ***0.85*** |  |  | ***0.46*** | ***11.56*** | ***6.32*** | ***0.07*** | ***0.07*** | ***0.13*** |
|  |  |  |  |  |  |  |  |  |  |  |  |  |  |  |  |
|  | 2 - BDS, 3 - TDS, 4 - QDS, M - Mane, N - Nocte, T - Tarde * - Controlled Release form | | | | | | | | | | | | | | |
