# Supplementary material for: Quantitative clinical assessment of motor function during and following LSVT-BIG® therapy
Source: J Neuroeng Rehabil. 2020 Jul 13;17:92. doi: 10.1186/s12984-020-00729-8 (PMC7359464; doi:10.1186/s12984-020-00729-8)
Supplement: Supplementary file 3 — Additional file 3 Table S3. Stages of assessment and the corresponding time points at which they occurred. [file 12984_2020_729_MOESM3_ESM.docx]

Table S3: Stages of assessment and the corresponding time points at which they occurred. Every subject adhered to the same data recording schedule.

| **Stage** | **Time of data recording session** |
| --- | --- |
| Pre1 | Monday morning, one week before therapy began |
| Pre2 | Monday morning, immediately before therapy session #1 |
| S4 | Monday morning, immediately before therapy session #5 (4 sessions complete) |
| S8 | Monday morning, immediately before therapy session #9 (8 sessions complete) |
| S15 | Thursday morning, immediately before therapy session #16 (15 sessions complete) |
| Post1 | Thursday afternoon, 2 weeks after final therapy session |
| Post2 | Thursday afternoon, 8 weeks after final therapy session |
| Post3 | Thursday afternoon, 13 weeks after final therapy session |
