# Supplementary figures and images for: Quantitative clinical assessment of motor function during and following LSVT-BIG® therapy
Source: J Neuroeng Rehabil. 2020 Jul 13;17:92. doi: 10.1186/s12984-020-00729-8 (PMC7359464; doi:10.1186/s12984-020-00729-8)

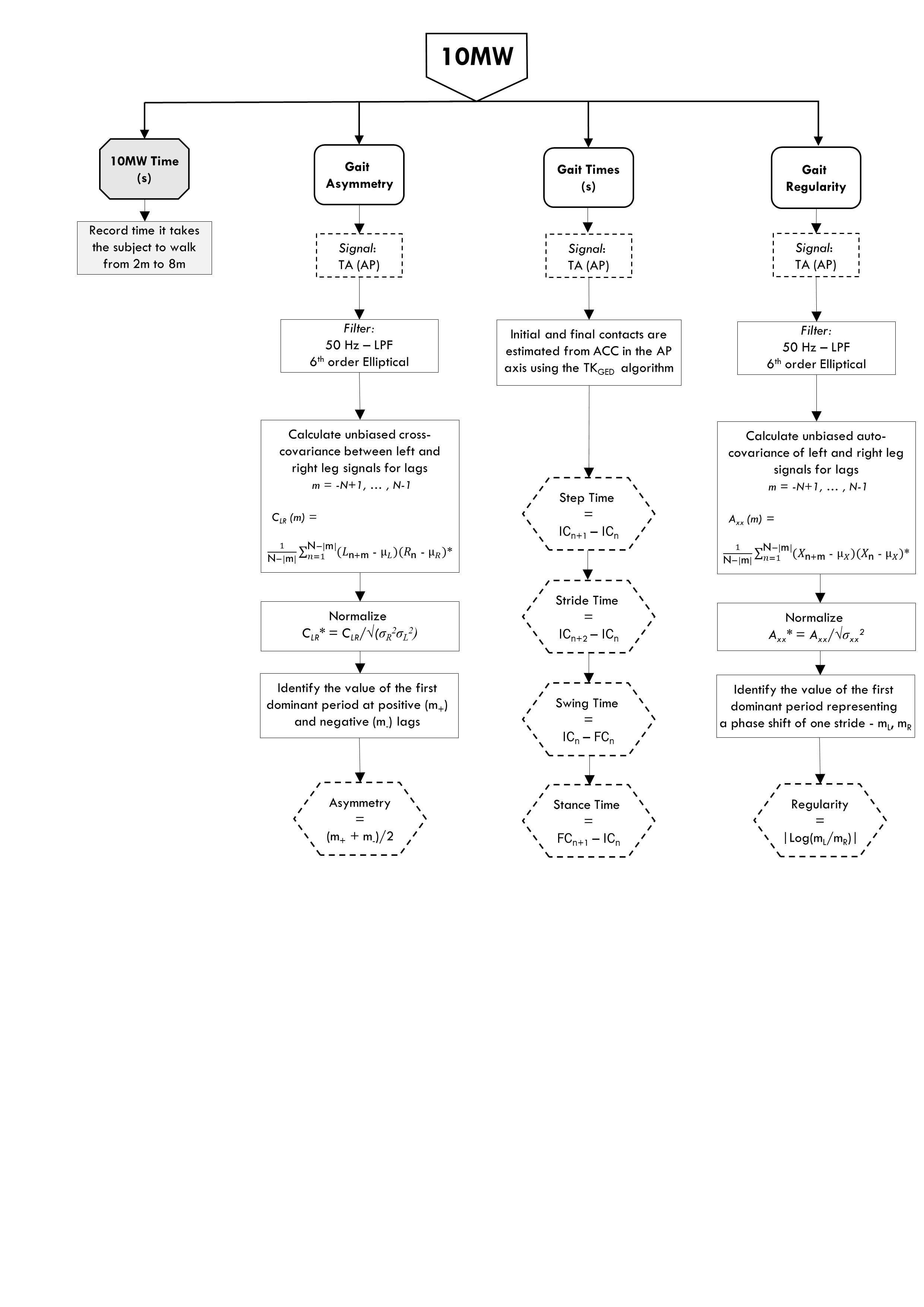

Supplement: Supplementary file 4 — Additional file 4 Fig. S1. Processing steps for ACC-measures calculated in i10MW task. [file 12984_2020_729_MOESM4_ESM.tif]

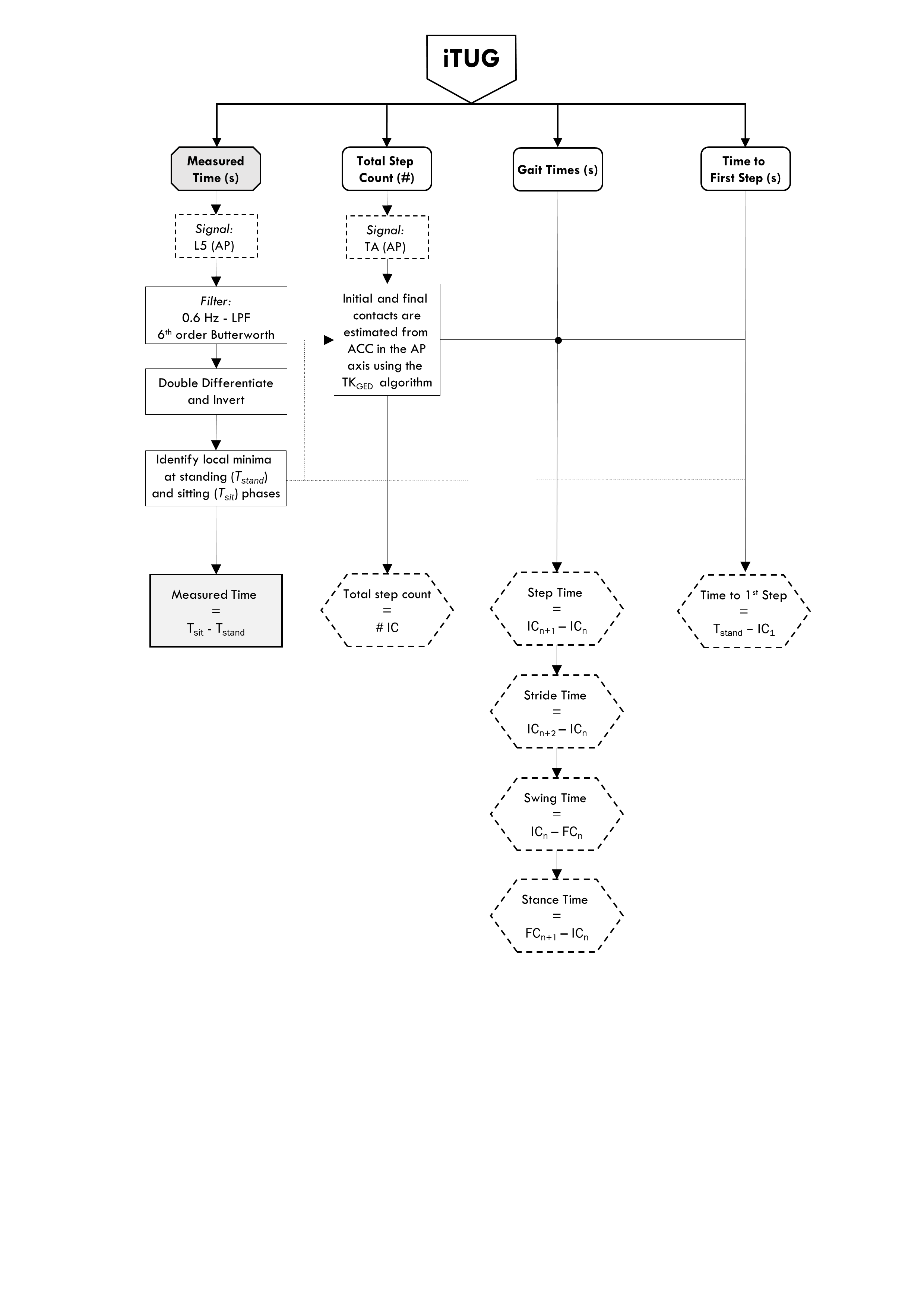

Supplement: Supplementary file 5 — Additional file 5 Fig. S2. Processing steps for ACC-measures calculated in iTUG task. [file 12984_2020_729_MOESM5_ESM.tif]

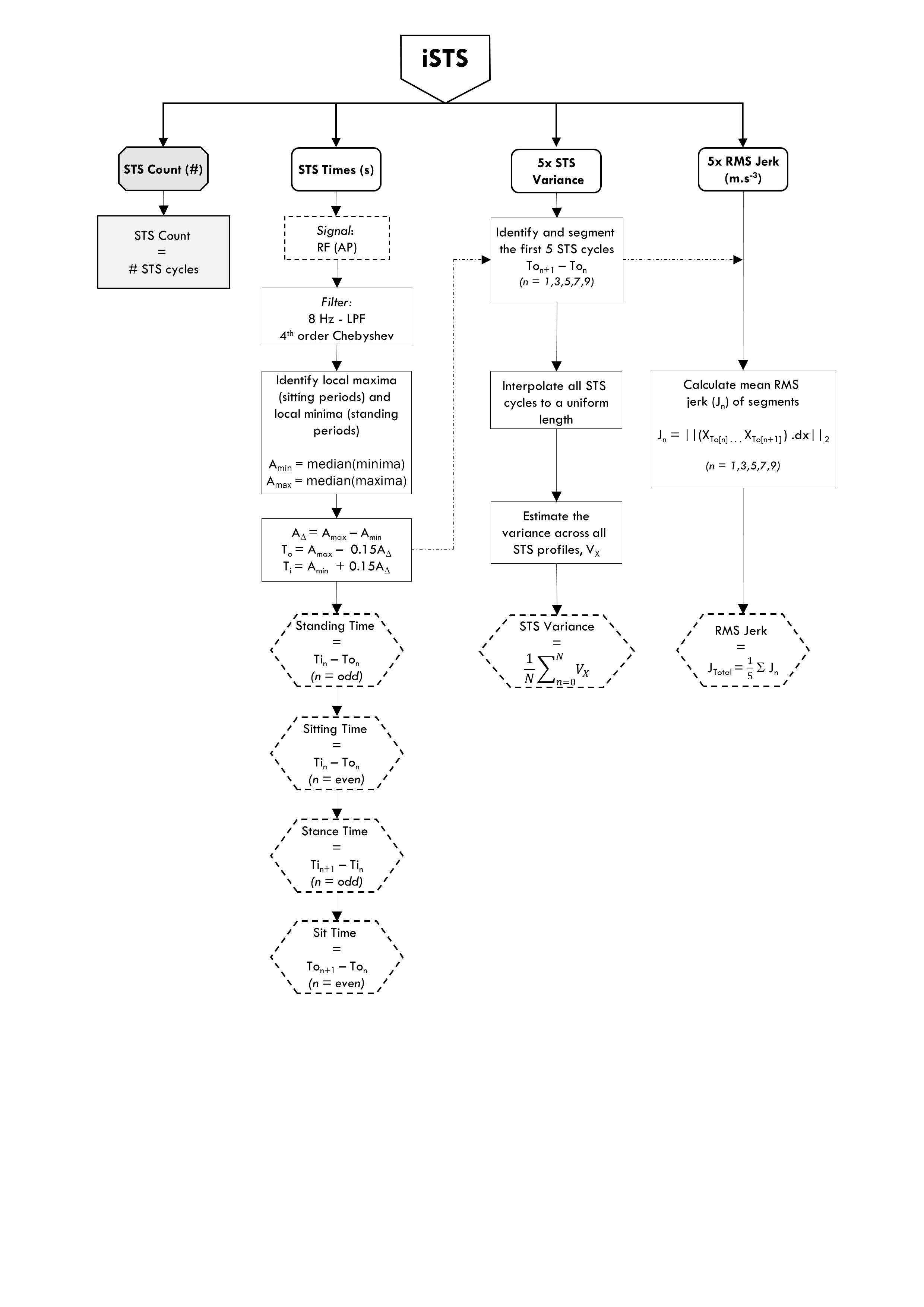

Supplement: Supplementary file 6 — Additional file 6 Fig. S3. Processing steps for ACC-measures calculated in iSTS task. [file 12984_2020_729_MOESM6_ESM.tif]

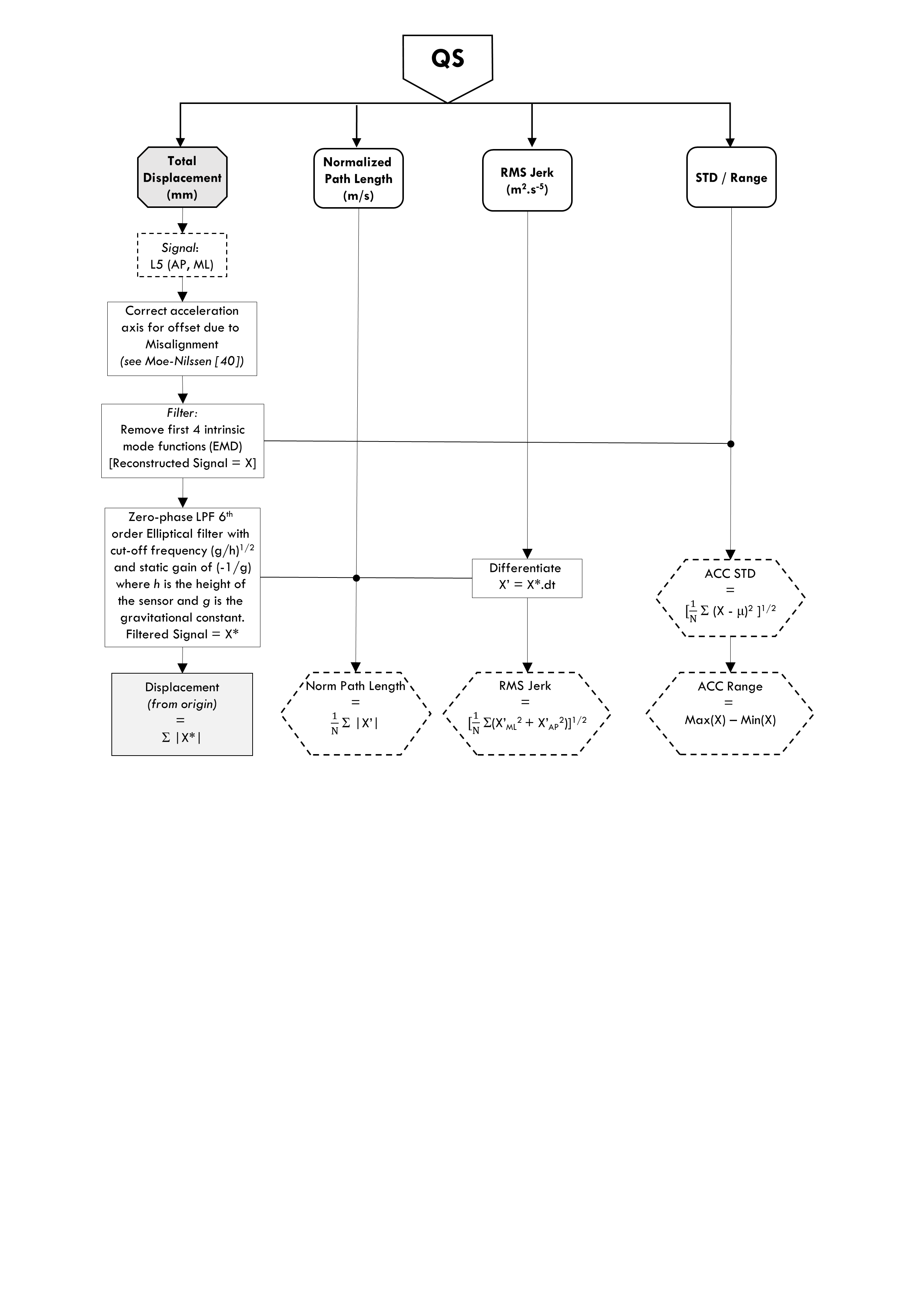

Supplement: Supplementary file 7 — Additional file 7 Fig. S4. Processing steps for ACC-measures calculated in QS task. [file 12984_2020_729_MOESM7_ESM.tif]

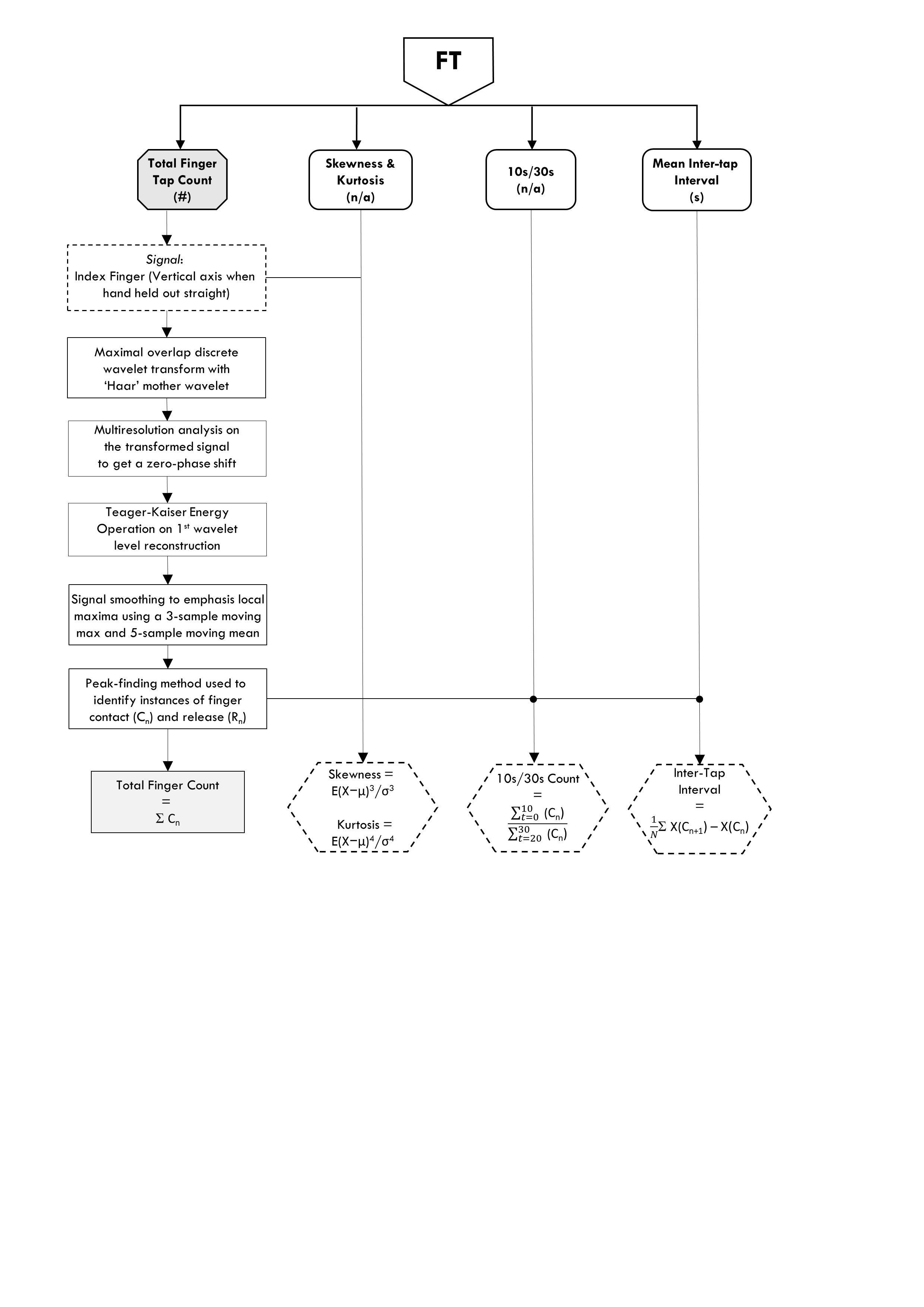

Supplement: Supplementary file 8 — Additional file 8 Fig. S5. Processing steps for ACC-measures calculated in FT task. [file 12984_2020_729_MOESM8_ESM.tif]
